# Making the grade: licensing examination performance by medical school accreditation status

**DOI:** 10.1186/s12909-022-03101-7

**Published:** 2022-01-14

**Authors:** Marta van Zanten, John R. Boulet, Christine D. Shiffer

**Affiliations:** 1 Foundation for Advancement of International Medical Education and Research (FAIMER), 3624 Market Street, 19104 Philadelphia, PA USA; 2 Educational Commission for Foreign Medical Graduates (ECFMG), Philadelphia, PA USA

**Keywords:** Accreditation, International medical schools, International medical graduates, Medical licensure examinations

## Abstract

**Background:**

Accreditation systems strive to ensure the quality of undergraduate (basic) medical education and encourage ongoing improvements. Despite increasing global emphasis on quality assurance activities, there is limited research linking accreditation of medical education to improved student and graduate outcomes. The purpose of this study is to compare the United States Medical Licensing Examination® (USMLE®) performance of students and graduates who attended international medical schools accredited by an agency recognized by the World Federation of Medical Education (WFME) to individuals who attended schools that did not meet this criterion.

**Methods:**

During the 2018-2020 study period, 39,650 individuals seeking Educational Commission for Foreign Medical Graduates® (ECFMG®) certification took one or more USMLE examinations. We cross-tabulated USMLE performance (first-attempt pass/fail result) and medical school accreditation status.

**Results:**

Individuals seeking ECFMG certification who attended international medical schools accredited by an agency recognized by WFME had higher or comparable USMLE first-attempt pass rates compared to individuals who attended medical schools that did not meet this criterion.

**Conclusions:**

Implementing and maintaining meaningful accreditation systems requires substantial resources. These results provide important positive evidence that external evaluation of educational programs is associated, on average, with better educational outcomes, including in the domains of basic science, clinical knowledge, and clinical skills performance.

## Background

In recent decades, rapid increases in the number of institutions delivering undergraduate (basic) medical education [1], along with dramatic growth in individuals migrating for educational and employment opportunities [2], and evolving patient diseases and demographics has intensified the global need for mechanisms that effectively evaluate the quality of medical instruction and training delivered [3, 4]. Accreditation systems help ensure that medical students have access to appropriate resources, are taught and assessed according to applicable standards, and are ready upon graduation to further their training or begin practice [5, 6]. For the purposes of this study, accreditation is defined as a process by which a designated authority, either a governmental entity or an independent body accountable at a governmental level, reviews and evaluates an educational program or institution on a cyclical basis [5]. In addition to mandating minimum standards, the accreditation process should encourage continuous institutional improvement and foster dissemination of effective educational practices, within countries and globally [5].

While accreditation systems around the world have similar goals, there are differences in the legislative mandates governing the systems, standards and processes employed, and the transparency and consequences of decisions [7]. Accreditation may be required by government or professional bodies or, depending on the jurisdiction, voluntarily requested by medical schools. Accreditation standards differ around the world in terms of the specific elements evaluated and the focus on process or defined outcomes. Accreditation protocols also vary globally, including the content of the self-study, rules defining qualifications of individuals who conduct the reviews, guidelines for the site visit, structure of accreditation reports, and procedures for making accreditation decisions. Outcomes of decisions can range from formative advice and support to immediate closure of schools that do not meet minimum standards [7].

### World Federation for Medical Education (WFME) Recognition Program

In response to this variability in accreditation systems worldwide and to support improved outcomes, the World Federation for Medical Education (WFME) implemented in 2010 a recognition program for medical school accrediting agencies with the goal of providing an independent, transparent, and rigorous method of ensuring that accreditation is conducted at an internationally accepted high standard [8]. As part of its recognition program, a WFME ad-hoc team evaluates the compliance of an accrediting agency against pre-defined criteria, including suitable governance structure and use of appropriate standards, processes, and procedures. The recognition process also includes a site visit, where the WFME team observes the agency performing an accrediting review of a medical school and attends an agency meeting where accreditation determinations are made regarding one or more other schools. The WFME team then writes a report describing the agency’s compliance with the WFME recognition criteria. The WFME Recognition Committee, a separate group, decides on agency recognition based on this report and associated evidence. WFME recognition is generally for 10 years and includes yearly monitoring. As of April 2021, 23 accrediting agencies functioning in 57 countries have been recognized by WFME and 16 agencies are in the process of recognition [8].

While other international systems exist that also evaluate accreditation protocols, such as the European Association for Quality Assurance in Higher Education (ENQA), the International Network for Quality Assurance Agencies in Higher Education Guidelines of Good Practice (INQAAHE/GGP) and the National Committee on Foreign Medical Education and Accreditation (NCFMEA), the WFME Recognition Program provides a comprehensive review focused specifically on evaluating the process of quality assurance of medical education. In addition to document evaluation and interviews with relevant individuals, the WFME program requires agencies use medical education relevant standards and includes direct observation of accreditation procedures [9].

### Educational Commission for Foreign Medical Graduates (ECFMG) Certification

While accreditation systems and the WFME Recognition Programme have an important role in ensuring the quality of medical education for domestic graduates around the world, these quality assurance processes are of growing importance in countries, such as the United States (US), with a large internationally educated healthcare workforce. Currently, almost one-quarter of doctors in residency training and in practice in the US are graduates of international medical schools (IMGs) [10]. IMGs play an important role in the US healthcare system: They are more likely than domestically educated physicians to practice primary care and fill positions in underserved areas [10–12]; they provide equal and, in some instances, better care than US graduates [13, 14]; and they make valuable contributions to diversity [15]. All IMGs must be certified by the Educational Commission for Foreign Medical Graduates (ECFMG) to enter accredited postgraduate medical education residency programs in the US, a prerequisite for unrestricted practice and licensure [16].

While there is general global consensus that accreditation of medical educational programs enhances learning experiences, there is limited research in this domain. Only a relatively small number of studies empirically demonstrate the association of accreditation with improved outcomes, such as increased student performance on examinations, e.g., the United States Medical Licensing Examination® (USMLE®) series or other national examinations [17–19]. Given the global rise in the number of accreditation systems and the increase in these agencies seeking to ensure the international credibility of their standards and processes through the WFME Recognition Programme, additional investigations are needed to demonstrate the linkage between these quality assurance efforts and improved outcomes. For example, a recent study of medical school factors associated with ECFMG certification compared ECFMG certification applicants from accredited versus non-accredited schools. Results showed that ECFMG certification rates, a measure of success in meeting stringent application, medical school, and examination requirements, were significantly greater for applicants who attended medical schools in countries with WFME-recognized agencies (16 at the time of the study) compared to applicants from countries without WFME-recognized agencies [20].

This current investigation expands on the Tackett et al. study [20] to include a broader study population of IMGs, incorporating data from the seven accrediting agencies that achieved WFME recognition since 2019, and additional outcome measures. The purpose of this study is to compare the 2018-2020 USMLE performance of ECFMG certification applicants who attended medical schools accredited by agencies recognized by WFME to applicants who attended non-accredited medical schools or medical schools accredited by an agency not recognized by WFME. Of note, this comparison encompasses the most recently available information regarding both USMLE performance and medical schools accredited by a WFME-recognized agency.

## Methods

### Study cohort

Our study cohort included 39,650 ECFMG applicants in the 2018-2020 time period. An applicant was defined as someone who registered and sat for an examination required for ECFMG certification. For this three-year cohort, 20,400 applicants were male (51.5%) and 19,250 were female (48.5%). The five largest groups by citizenship at entry to medical school were the United States; (n=10,292, 26.0%), India; (n=5,214, 13.2%), Canada; (n=2,382, 6.0%), Pakistan; (n=2,322, 5.8%), and Nigeria; (n=1,197, 3%). The remaining applicants indicated citizenship from an additional 171 countries. The top five countries of medical education for this group of applicants were India; 12.8% (n=5,093), Grenada; 9.0% (n=3,547), Pakistan; 6.5% (n=2,561), Barbados; 3.8% (n=1,505), and China; 2.7% (n=1,070). The remaining applicants attended medical schools located in 153 additional countries. While 60.7% (n=24,065) of applicants indicated that English was not their native language, 81.6% (n=32,338) reported attending a medical school with some or all instruction in English.

### United States Medical Licensing Examination (USMLE)

Amongst other requirements, up until March 2020, the ECFMG certification process required successful completion of three components of the USMLE: Step 1 Basic Science; Step 2 Clinical Knowledge (CK); and Step 2 Clinical Skills (CS) [16]. Administration of Step 2 CS was suspended in March 2020 due to the COVID-19 pandemic and has since been permanently discontinued [21]. Step 1 and Step 2 CK are multiple-choice tests, while Step 2 CS was a performance-based examination. As part of Step 2 CS, physician examinees interacted with 12 standardized patients (SPs) as they would with actual patients, gathering and sharing relevant information, performing a focused physical examination, and writing up their findings in clinical notes. Step 2 CS was divided into three conjunctive scoring subcomponents: Integrated Clinical Encounter (ICE); Communication and Interpersonal Skills (CIS); and Spoken English Proficiency (SEP). The ICE portion comprised data-gathering skills (history taking and performing a relevant physical examination), scored by SPs who completed case-specific checklists, and written summary notes (synthesis of data gathered and written communication), scored by trained physician raters. CIS was scored by the SPs completing case-specific checklists; SEP was scored globally by the SPs. To pass Step 2 CS, examinees were required to achieve passing scores on all three subcomponents (ICE, CIS, and SEP) in the same administration. While the USMLE components can be taken in any order, most physicians seeking ECFMG certification took Step 1 first, followed by Step 2 CK, and lastly Step 2 CS.

### Medical school accreditation

In June 2020, the staff of ECFMG began compiling a list of all medical schools worldwide accredited by a WFME-recognized agency, using publicly available information from agencies’ websites and similar sources, including in some cases verification directly with the agency. Importantly, to be included in the list, the school must have already met ECFMG requirements, meaning that its students and graduates were currently eligible for ECFMG certification. Among its criteria, ECFMG requires that the medical school be recognized by the appropriate government authority in the country where the school is located, and that graduates of the school are eligible to practice medicine in that country [16]. Accreditation is not required unless required by the appropriate government authority for recognition of the medical school. Over several months, schools were added to the list as they received accreditation or had their accreditation status confirmed. The final list used in this study was updated as of November 2, 2020 and encompassed 559 schools.

### Analysis

Using the cohort of ECFMG applicants described earlier, we cross-tabulated USMLE performance (first-attempt pass/fail result) and medical school accreditation status. We chose first-attempt performance because it allows for meaningful comparisons across the three examinations. We did not have access to the USMLE scores. However, for Step 2 CS we had pass/fail status for all three subcomponents (ICE, CIS, SEP). For this examination, we were able to cross-tabulate overall and subcomponent performance (pass/fail) with accreditation status. For all analyses, we compared first-attempt pass rates by accreditation status (accredited by an agency recognized by WFME versus not accredited or accredited by a non-WFME recognized agency). Given that our data was the population of ECFMG applicants from 2018 to 2020, no inferential statistics were calculated.

### Ethical Considerations

As part of the ECFMG Application for Certification, individuals must complete a notarized Certification of Identification and provide demographic information. The ECFMG privacy policy [22] indicates that ECFMG reserves the right to aggregate data provided by individuals for quality assurance purposes and on-going research in accordance with our mission. Individuals acknowledge that their data can be used for research, or their records are not included in any analysis. The final data set was fully anonymized and it is not possible to trace any data back to individual physicians. Medical school accreditation and agency recognition statuses were obtained from publicly available sources [23].

## Results

Across the three-year study period (2018 – 2020), 39,650 individuals took one or more examinations required for ECFMG certification: 36,828 applicants took USMLE Step 1, 17,860 applicants took Step 2 CK and 10,345 took Step 2 CS. First-attempt pass rates by accreditation status of the applicant’s medical school are presented in Table 1 (Step 1 and Step 2 CK) and in Table 2 (Step 2 CS overall, ICE, CIS and SEP). For all USMLE components and subcomponents, individuals who attended medical schools accredited by an agency recognized by WFME (“recognized medical schools”) had higher first-attempt pass rates compared to individuals who attended medical schools that did not meet this criterion, either because the school was not accredited or the school was accredited by an agency not recognized by WFME (“non-recognized medical schools”). Of the three examinations, the difference was greatest for Step 1, with 88.4% of individuals from recognized medical schools passing on their first attempt, compared to 78.6% from non-recognized medical schools. For Step 2 CK, first-attempt pass rates were 91.8% versus 88.8%, respectively. For Step 2 CS, 81.8% of individuals who attended recognized medical schools passed the exam on the first attempt, compared to 73.8% of their peers from non-recognized schools.

**Table 1:** First-attempt pass rates on USMLE Step 1 and Step 2 CK by recognition status of medical school attended by ECFMG applicant for certification

	Step 1 Applicants, No. (%)			Step 2 CK Applicants, No. (%)		
	Recognized^a^	Non-recognized^b^	Total	Recognized^a^	Non-recognized^b^	Total
Pass	13,409 (88.4)	17,019 (78.6)	30,428	6,781 (91.8)	9,296 (88.8)	16,077
Fail	1,752 (11.6)	4,648 (21.5)	6,400	610 (8.3)	1,173 (11.2)	1,783
Total	15,161	21,667	36,828	7,391	10,469	17,860

^a^attended a medical school accredited by an agency recognized by the World Federation for Medical Education (WFME)

^b^attended a medical school not accredited or accredited by an agency not recognized by WFME

**Table 2:** First-attempt pass rates on USMLE Step 2 CS overall and on ICE, CIS and SEP subcomponents by recognition status of medical school attended by ECFMG applicant for certification

	Step 2 CS Overall Applicants, No. (%)			Step 2 CS ICE Applicants, No. (%)			Step 2 CS CIS Applicants, No. (%)			Step 2 CS SEP Applicants, No. (%)		
	Recognized^a^	Non-recognized^b^	Total	Recognized^a^	Non-recognized^b^	Total	Recognized^a^	Non-recognized^b^	Total	Recognized^a^	Non-recognized^b^	Total
Pass	3,038 (81.8)	4,893 (73.8)	7,931	3,218 (86.7)	5,288 (79.8)	8,506	3,562 (95.9)	6,330 (95.5)	9,892	3,543 (95.4)	6,134 (92.5)	9,677
Fail	675 (18.2)	1,739 (26.2)	2,414	495 (13.3)	1,343 (20.3)	1,838	151 (4.1)	301 (4.5)	452	170 (4.6)	497 (7.5)	667
Total	3,713	6,632	10,345	3,713	6,631	10,344	3,713	6,631	10,344	3,713	6,631	10,344

^a^attended a medical school accredited by an agency recognized by the World Federation for Medical Education (WFME)

^b^attended a medical school not accredited or accredited by an agency not recognized by WFME

For Step 2 CS, we also compared pass rates for the three subcomponents (ICE, CIS, SEP) by the accreditation status of an individual’s medical school. The pass rate difference was greatest for ICE, with 86.7% of individuals from recognized schools passing the subcomponent on the first attempt, compared to 79.8% for individuals from non-recognized schools. There was a negligible difference with respect to recognition status on first-attempt pass rates for the CIS component (95.9% versus 95.5%). For SEP, 95.4% of individuals from recognized medical schools passed the language subcomponent on their first attempt, while 92.5% of their non-recognized school peers passed it on their first attempt.

## Discussion

The purpose of this study was to investigate the relationship between accreditation of medical schools by a WFME-recognized agency and students’ and graduates’ examination performance. While accreditation systems are generally viewed as driving quality, such systems are resource intensive and require ongoing stakeholder support. Given these demands, and the growth of accreditation systems worldwide, evidence is needed to support the validity of medical education accreditation activities by conducting investigations that tie accreditation to improved outcomes. Our study also attempted to lend support to the WFME process of evaluating accrediting agencies to ensure these quality assurance processes are functioning at an acceptable standard.

Individuals who attended medical schools accredited by a WFME-recognized agency demonstrated better or comparable performance on all measures used in this analysis, USMLE Step 1, Step 2 CK and Step 2 CS overall, and subcomponents of Step 2 CS (ICE, CIS and SEP). These results are important because strong examination performance is one of many potential outcomes associated with higher quality physicians [14, 24–27]. For example, studies have demonstrated that Step 2 CK was predictive of specialty board certification examination scores and certification status [28], and Step 2 CS scores are related to residency performance [26, 29, 30]. Stakeholders generally support the appropriateness of the USMLE cut scores used to make classification decisions [31]. While it is not possible in this study to establish the causal mechanisms that lead to greater licensure examination performance, it remains that those students attending or graduating from recognized accredited medical schools outperform, at least on average, those who do not.

Of the three USMLE components (Step 1, Step 2 CK and Step 2 CS), the largest differences in first-attempt pass rates by recognized accreditation status of the medical school were found with performance on Step 1, followed by Step 2 CS, and Step 2 CK. These findings are consistent with previous studies investigating the associations between medical school accreditation and student outcomes [20, 32–34]. Reasons for the variations in the size of the differences in pass rates for the licensure examinations we studied may rest with their specific content and focus. For Step 1, which tests basic science knowledge, many accreditation standards are directly related to aspects of the preclinical phase of education, such as ensuring the hiring of qualified teachers and the provision of robust laboratory experiences. Medical schools that are adequately resourced to deliver basic science education are likely to obtain a positive accreditation status and the students would be expected to perform well on examinations covering these knowledge domains. In addition, admission criteria may differ between accredited and non-accredited schools, with accredited schools preferentially selecting students who score higher on pre-admission tests that generally reflect superior prior knowledge of basic sciences.

Compared with Step 1, accreditation standards may have less direct impact on student performance on clinical examinations because students’ experiences in the clinical phase of their education are likely more varied. Nevertheless, the greater likelihood of passing Step 2 CS for those attending recognized accredited institutions is noteworthy because the purpose of this examination is to evaluate a physician’s skills in a “real-world,” albeit simulated, setting. Here, the performance difference by recognition status may be a function of accreditation standards that are related to the clinical training phase of education, such as access to clinical simulation technology and ensuring adequate patient exposure experiences. Here, one would expect that recognized accredited schools would provide better clinical training. Of the three subcomponents of Step 2 CS (ICE, CIS, SEP), the performance difference (% passing on 1st attempt) by recognition status was greatest for ICE, followed by SEP, and CIS. The ICE subcomponent measures a physician’s ability to gather and summarize patient data, skills directly related to accreditation standards that evaluate the overall quality of clinical medical education. The CIS and SEP subcomponents, while still positively associated with recognized accreditation, are related to a physician’s personal ability to tactfully and empathetically interact with patients and speak English clearly and fluently. These personal characteristics and behaviors are certainly important for medical students but less specifically tied to accreditation standards.

The results of this current investigation, combined with similar findings from previous studies [20, 32–34], lend support to progressing accreditation and WFME recognition beyond a voluntary endeavor. For example, to stimulate international accreditation activities and improve the quality of medical education worldwide, starting in 2024, applicants for ECFMG certification will be required to be a student or graduate of a medical school accredited by a WFME-recognized agency. The requirement is intended to encourage the development and implementation of standards for evaluating undergraduate medical education, to provide greater assurance to both medical students and the public that they will be appropriately trained [35, 36]. For international medical students seeking residency training positions in the US, ECFMG certification is required. Given that passing USMLE is part of the certification process, and those who attend recognized accredited schools are more likely to pass on first attempt, stakeholders can make more informed decisions about where to obtain quality education.

While our results generally showed support for accreditation and the WFME Recognition Programme, there are a number of important factors to consider when interpreting these results. We conducted a cross-sectional study and therefore the results should not be interpreted as support for causation. First, because no pre-medical school variables were available for analysis, the prior ability level of individuals and the relationship between an individual’s choice of medical school (accredited or not) is unknown. Second, international medical schools may provide differing levels of preparation for the USMLE which may or may not be related to accreditation status. Third, better schools may be more likely to seek accreditation, and also attract higher quality students. Fourth, while schools were designated as recognized accredited at the time of our study, some individuals may have attended schools one or two years prior to the school receiving a positive accreditation status. However, an accreditation decision date could be viewed as an approximate marker for quality verification, as schools likely enhance resources and educational procedures for several months or years in preparation for accreditation. Finally, individuals who take USMLE and pursue ECFMG certification are a self-selected group and their performance may not reflect the overall quality of students who attend their school and the relationship to the school’s accreditation status.

Variables associated with the recognition status of a medical school could also potentially impact the results. The list of recognized schools was created using a manual process and only included schools that had already met ECFMG requirements. Therefore, the list used does not encompass the full universe of schools accredited by a WFME-recognized agency. In addition, the prevalence of COVID-19 in many countries prohibited some schools from achieving accreditation during the time the list was compiled. The pandemic has also paused WFME Recognition Programme site visits and numerous agencies are awaiting review [23]. Including student performance from schools accredited by agencies that have increased the quality of their systems in anticipation of WFME recognition would enlarge the amount of data available for analysis, and therefore could strengthen potential associations.

While accreditation may be voluntary or mandatory, depending on the jurisdiction of the medical school, participation by the accrediting agency in the WFME Recognition Programme is voluntary. Given the future ECFMG 2024 requirement, agencies that accredit medical schools attracting large numbers of students who seek ECFMG certification may have been more motivated to undergo the WFME review. Despite this potential external push for recognition, and lending support to accreditors’ aspirations to raise medical education quality in all their country’s medical schools, the 23 agencies currently recognized by WFME are geographically diverse and located in countries with a range of population and income levels. Moreover, many are located in countries that historically do not produce large numbers of migrating physicians [23]. While these medical school and accrediting agency variables may potentially impact the quality of students in attendance, the consequential impact of accreditation, and the recognition of the accreditor, remains positive.

While our results support the use of robust accreditation systems to increase physician quality, at least in terms of licensure examination performance, further studies are warranted. The relationship between accreditation status and student outcomes could be strengthened by examining performance of students from accredited schools versus performance of students from schools that attempted accreditation but were denied. Tracking student performance data over time as a school achieves accreditation and makes accreditor-mandated improvements would provide additional meaningful evidence supporting quality assurance systems. Investigating the differential impact of elements of accreditation systems, such as the use of quality improvement markers [37], may help substantiate various protocols. Most importantly, investigations of the relationships between accreditation of an individual’s medical school and subsequent direct patient care data is needed to further support accreditation efforts and the WFME Recognition Programme results.

## Data Availability

The list of medical schools accredited by an agency recognized by the World Federation for Medical Education (WFME) and eligibility of graduates to apply for Educational Commission for Foreign Medical Graduates (ECFMG) certification is available here: https://www.ecfmg.org/certification-requirements-2022-match/pathways-3-5.html (accessed 19 May 2021)Data is available from the corresponding author upon reasonable request.

## Abbreviations

CIS: Communication and Interpersonal Skills
ECFMG: Educational Commission for Foreign Medical Graduates
ENQA: European Association for Quality Assurance in Higher Education
INQAAHE/GGP: International Network for Quality Assurance Agencies in Higher Education Guidelines of Good Practice
FAIMER: Foundation for Advancement of International Medical Education and Research
IMGs: International Medical Graduates
ICE: Integrated Clinical Encounter
NCFMEA: National Committee on Foreign Medical Education and Accreditation
SEP: Spoken English Proficiency
USMLE: United States Medical Licensing Examination
CK: USMLE Step 2 Clinical Knowledge
CS: USMLE Step 2 Clinical Skills
WFME: World Federation for Medical Education

## Declarations

All methods were carried out in accordance with relevant guidelines and regulations.
